# Impact of a constructivist anesthesia education model on preoperative anxiety and perioperative outcomes in laparoscopic cholecystectomy

**DOI:** 10.3389/fmed.2025.1633718

**Published:** 2025-10-08

**Authors:** Yan Liang, Peng Wang, Haodan Zhao, Biying Li, Jiegang Zhao, Hui Sun, Lin Wang, Fuqiang Xing

**Affiliations:** ^1^The First People's Hospital of Luoyang, Luoyang, China; ^2^The First Affiliated Hospital of Henan University of Science and Technology, Luoyang, China

**Keywords:** constructivism, anesthesia, anxiety, health education, nursing, clinical trial

## Abstract

**Introduction:**

This study aimed to evaluate the effectiveness of a constructivist-based anesthesia education model in reducing preoperative anxiety and improving perioperative outcomes among patients undergoing laparoscopic cholecystectomy.

**Methods:**

A total of 106 patients from a tertiary hospital in Luoyang, China, were enrolled and divided into an intervention group, which received the anesthesia education program, and a control group, which received conventional preoperative education. The intervention, delivered over one week, included micro-videos, interactive simulations, and guided exercises designed to actively engage patients. Anxiety levels were assessed using the State Anxiety Inventory (SAI), while anesthesia knowledge was evaluated through a structured questionnaire. Physiological parameters, including blood pressure and heart rate, were measured at various perioperative time points.

**Results:**

The results showed that the intervention group had significantly lower SAI scores compared to the control group after the intervention (39.06 ± 3.08 vs. 41.64 ± 7.55, *p* < 0.05) and higher anesthesia knowledge scores (88.21 ± 10.23 vs. 81.37 ± 11.66, *p* < 0.05). Additionally, the intervention group exhibited improved physiological stability during operating room admission and anesthesia recovery, with significant reductions in blood pressure and heart rate (*p* < 0.05).

**Discussion:**

These findings demonstrate that the constructivist education model effectively reduces preoperative anxiety, enhances anesthesia knowledge, and optimizes perioperative physiological responses. This approach addresses the psychological and informational needs of surgical patients, offering a scalable framework for improving perioperative care and patient outcomes in diverse clinical settings.

## 1 Introduction

Preoperative anxiety is a prevalent psychological disorder among surgical patients, particularly those undergoing laparoscopic cholecystectomy. This anxiety often arises from fears about anesthesia, surgical outcomes, and the unknowns of the perioperative experience, negatively affecting both psychological wellbeing and physiological stability. Studies indicate that approximately 36.61% of surgical patients experience preoperative anxiety, which is linked to elevated blood pressure, increased heart rate (HR), impaired immune function, and delayed wound healing. These physiological effects not only heighten the risk of perioperative complications but also demand increased use of anesthetic and analgesic agents, complicating postoperative recovery (1–4).

Effective preoperative education has long been recognized as a strategy to reduce anxiety and improve patient outcomes. Traditional methods, such as verbal instructions and printed materials, have evolved into multimedia and web-based platforms. However, these approaches often fail to engage patients actively or address their psychological and emotional needs, underscoring the necessity for more innovative, patient-centered educational strategies (5–7).

Constructivism learning theory offers a compelling framework for addressing these challenges. Unlike traditional, didactic teaching methods, constructivism emphasizes active participation, contextual learning, collaboration, and meaning-making. Learners, according to this theory, acquire knowledge through personalized and interactive experiences that allow for deeper internalization (8, 9). In healthcare, this approach can empower patients to take an active role in their care, enhancing their understanding of medical procedures and fostering collaboration with healthcare providers.

While constructivism has been widely applied in general education and professional training settings, its use in healthcare, particularly in surgical education, remains relatively unexplored. Most existing studies have focused on formal classroom environments, with limited research addressing how these principles can be adapted to preoperative patient education. Specifically, there is a gap in applying constructivist approaches to meet the unique needs of surgical patients, such as managing preoperative anxiety, understanding anesthesia, and preparing for surgery (10).

This study aimed to fill that gap by developing an anesthesia health education model based on constructivist principles, specifically focusing on patients undergoing laparoscopic cholecystectomy. In contrast to traditional education methods, this model could incorporate interactive, experiential components, such as micro-videos and simulation-based training, which are designed to engage patients actively and help them internalize main concepts. By addressing both informational and emotional needs, the model could reduce preoperative anxiety and improve both physiological and psychological outcomes in the perioperative period. The findings of this study may provide valuable insights into the application of constructivism-based education in clinical practice. By bridging the existing gap in surgical education research, this approach has the potential to set a new standard for preoperative patient education, optimize perioperative care, and improve patient outcomes in diverse clinical settings.

## 2 Methods

### 2.1 Subjects

A convenience sampling method was used to recruit patients scheduled for laparoscopic cholecystectomy at a tertiary hospital in Luoyang. This approach was used due to the nature of the patient population available at the hospital during the study period. It was selected to ensure a sufficient sample size for analysis within a feasible timeframe, while maintaining the study’s inclusion criteria. The inclusion criteria were: (1) patients undergoing general anesthesia, (2) ASA classification Grade I or II, (3) age between 18 and 75 years, (4) clear consciousness with normal communication and comprehension abilities, and (5) voluntary participation in the study. Exclusion criteria included: (1) patients admitted directly to the operating room from emergency or outpatient departments, (2) patients with a history of previous surgery or anesthesia, and (3) patients with an ASA classification III or higher.

While this study provides valuable insights into the effects of anesthesia health education on anxiety and knowledge in lower-risk patients, the exclusion of high-risk (ASA III+) patients may limit the generalizability of these findings to populations with more complex comorbidities or those undergoing emergent surgeries. In such populations, anxiety levels, educational needs, and cooperation during the perioperative period may differ significantly, potentially influencing both the outcomes of the intervention and patient responses. Future studies should consider including these higher-risk groups to assess the broader applicability of the intervention.

The sample size was calculated using the formula: *N*1 = *N*2 = 2[(t*α*/2 + t*β*)*S*/*δ*]2. With *α* = 0.05, *β* = 0.1, preliminary experiments yielded *σ* = 3.523 and *δ* = 2.497, resulting in *δ*/*σ* ≈ 0.7. Substituting these values, the required sample size was *N*1 = *N*2 = 48. To account for potential dropout, the sample size was increased by 10%, resulting in 53 patients per group and a total of 106 participants. The control and intervention groups were recruited consecutively, with patients assigned to the control group from July to August 2024 and those assigned to the intervention group from September to October 2024. This consecutive recruitment process was implemented to avoid time-related biases that might arise from simultaneous recruitment, ensuring a clear separation between the two groups. Given the use of temporal grouping (July–August vs. September–October), the potential for seasonal or temporal biases, such as staffing variations, changes in hospital protocols, or other environmental factors, was considered. To mitigate these biases, several strategies were used. First, the study was conducted within a defined, short timeframe, and all interventions were standardized to ensure consistency. The same multidisciplinary research team conducted all educational sessions and anesthesia procedures, ensuring uniformity across the two groups. Furthermore, the hospital’s protocols and staffing remained largely consistent throughout the study period, and any seasonal variations that could impact the outcomes, such as staffing shortages or changes in anesthesia procedures, were monitored. In addition, statistical methods, including randomization and within-group comparisons, were used to account for potential confounding variables. These steps helped reduce the influence of temporal factors and ensured that observed differences between the groups were due to the intervention rather than external confounding variables. The study received approval from the Ethics Committee of The First People’s Hospital of Luoyang (Ethics Code: 2024123004). Written informed consent was obtained from all participants prior to enrollment. All collected data were anonymized to protect patient confidentiality. Personal identifiers were removed, and a unique study identification code was assigned to each participant. All data were securely stored in compliance with institutional privacy regulations and accessed only by authorized personnel involved in the research.

### 2.2 Intervention

#### 2.2.1 Control group

The control group received routine preoperative health education, which included fasting instructions, preoperative preparation processes, an introduction to the operating room environment, anesthesia methods, cooperation requirements, postoperative transfer processes, and instructions for using the patient-controlled analgesia (PCA) pump.

#### 2.2.2 Intervention group

The intervention group received a constructivism learning theory-based anesthesia health education program tailored to laparoscopic cholecystectomy patients.

#### 2.2.3 Intervention protocol

##### 2.2.3.1 Research team establishment

A multidisciplinary team was formed, comprising one anesthesiologist, two anesthesia nurses, two hepatobiliary surgical nurses, and two master’s students in nursing. Anesthesia nurses delivered anesthesia health education, while master’s students and surgical nurses collected and organized data. The anesthesiologist provided expert guidance on anesthesia-related content. To ensure consistency and reliability in data collection and intervention delivery, all personnel involved in the study underwent training sessions. These sessions included calibration exercises for delivering the educational program, as well as guidance on data collection procedures. A pre-study meeting was held to standardize the protocol and ensure that each team member was familiar with the intervention components, data recording forms, and the overall study protocol.

##### 2.2.3.2 Pre-intervention preparation

###### 2.2.3.2.1 Micro-video development

A three-part micro-video series was created through scriptwriting, role-playing simulations, video recording, and post-production editing using Adobe Premiere Pro CS3. The video featured ward nurses, surgeons, anesthesiologists, anesthesia nurses, patients, family members, operating room receptionists, and recovery room staff. The content was divided into three segments: the day before anesthesia, the operating room process, and postoperative ward return. It covered topics such as pre-anesthesia precautions, operating room environment, anesthesia cooperation, and postoperative instructions. The total video length was 8 min.

##### 2.2.3.3 Intervention implementation

**Figure 1:**
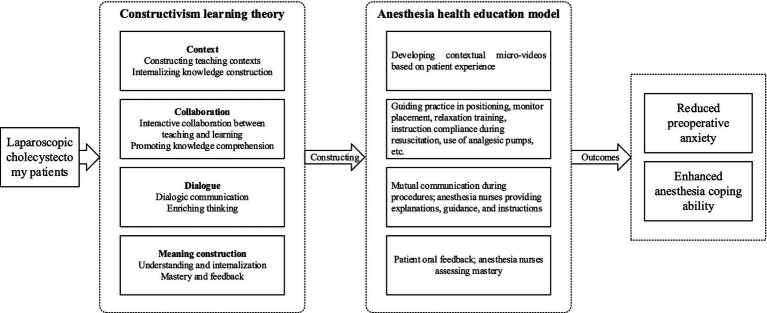
Framework diagram of constructivism learning theory model used in this study.

The intervention group received a preoperative anesthesia health education program explicitly designed based on constructivism learning theory (Figure 1). This model emphasizes active participation, experiential learning, and the construction of knowledge through interactive and contextualized experiences. In comparison to other active learning frameworks, such as cognitive-behavioral interventions, the constructivism approach fosters a deeper understanding by encouraging patients to actively engage in hands-on exercises and self-reflection. Unlike cognitive-behavioral methods that often focus on changing thought patterns or behaviors through structured strategies, the constructivism model prioritizes the patient’s role in actively constructing knowledge based on their experiences. This approach promotes long-term retention and comprehension, as it involves patients in real-world simulations of their perioperative experiences.

Furthermore, while cognitive-behavioral interventions typically focus on reducing anxiety through cognitive restructuring and stress management techniques, the constructivist model in this study integrates anxiety-reduction strategies, such as relaxation exercises, within the context of hands-on learning. This contextualized learning enhances patient engagement and encourages them to link their learning experiences with practical, real-world applications. This is especially valuable in preparing patients for complex processes, such as anesthesia, where patient understanding and cooperation are critical for procedural success and overall recovery.

### 2.3 Tools

#### 2.3.1 State Anxiety Inventory

The State Anxiety Inventory (SAI) was used to assess patients’ immediate anxiety levels. Administered on the day before surgery, both pre- and post-intervention, the scale consisted of 20 multiple-choice questions scored on a 4-point Likert scale (1–4 points), with total scores ranging from 20 to 80. Higher scores indicated higher anxiety levels. While a significant statistical difference in SAI scores was observed between the two groups (39.06 vs. 41.64), the minimal clinically important difference (MCID) for SAI in this population was referenced to evaluate the clinical relevance of this change. In this study, the MCID threshold for the SAI was determined based on previously published data for similar populations undergoing laparoscopic surgeries, where a difference of 5 points was considered clinically significant. The observed reduction in the intervention group (2.58 points) did not reach this threshold, suggesting that while statistically significant, the clinical relevance of the reduction in anxiety levels may be modest.

#### 2.3.2 Anesthesia health knowledge questionnaire

This questionnaire, based on Han Xiaojuan’s Delphi expert consultation framework, assessed patients’ knowledge of anesthesia health education. It covered four dimensions: pre-anesthesia precautions, anesthesia cooperation, cooperation before extubation, and postoperative analgesia. Scores ranged from 0 to 100, with higher scores reflecting greater knowledge.

#### 2.3.3 Biological parameters

**Figure 2:**
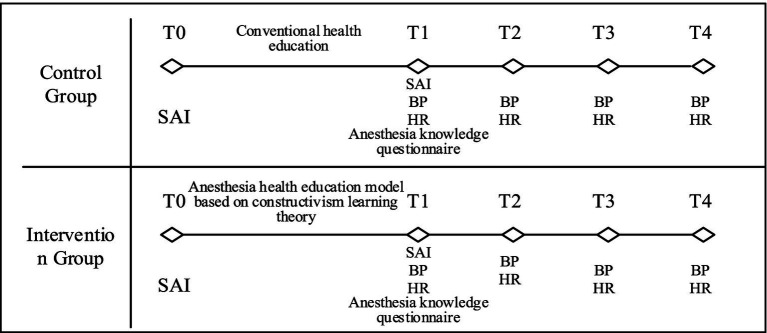
Data collection diagram. T0: 1 day before surgery, pre-intervention; T1: 1 day before surgery, post-intervention; T2: upon entering the operating room; T3: at anesthesia recovery; T4: after extubation; and T5: 3 days post-surgery.

Physiological parameters, including systolic blood pressure (SBP), diastolic blood pressure (DBP), and HR, were recorded at six points: T0: 1 day before surgery (pre-intervention), T1: 1 day before surgery (post-intervention), T2: upon entering the operating room, T3: during anesthesia recovery, T4: after extubation, and T5: 3 days post-surgery. Post-extubation measurements (T4) were also influenced by sedation or analgesia administration, which may have contributed to the non-significant differences observed in SBP and DBP at this time point. Figure 2 presents a detailed timeline of data collection.

### 2.4 Quality control

Standardized equipment was used for all measurements, including the Mindray BeneVision N17 monitor and Dräger Primus anesthesia machine. The same anesthesiologist performed anesthesia induction and monitoring for all patients, while the same anesthesia nurse conducted health education sessions for consistency. To ensure quality and reduce variability in outcomes, the following control measures were implemented:

1. *Sedation control:* all patients underwent a standardized anesthesia protocol, with the same anesthesiologist managing anesthesia induction, maintenance, and emergence. Postoperative sedation levels were closely monitored as part of routine care to ensure they remained within expected ranges and did not significantly affect post-extubation outcomes.
2. *Pain control:* pain management was standardized across both groups through the use of patient-controlled analgesia (PCA) pumps. Pain levels were monitored using the Numerical Rating Scale (NRS) at regular intervals. Appropriate interventions were made to maintain effective pain control, minimizing variability that might impact physiological parameters, such as HR and blood pressure.

Although residual sedation and unmeasured pain could potentially explain the differences in post-extubation HR (which remained significantly different between groups) and SBP/DBP (which did not), their direct effects were not quantified in this study. Future research should include direct monitoring of sedation and pain levels to better elucidate their role in post-extubation physiological variations. To ensure the accuracy and reliability of the data, a two-step verification process was used. This included double data entry and independent verification by two trained researchers to minimize errors and ensure consistency.

### 2.5 Statistical analysis

Statistical analysis was conducted using SPSS 23.0 software (IBM, Armonk, NY, USA). Normally distributed data were expressed as mean ± standard deviation, and independent sample t-tests and repeated-measure ANOVA were used for comparisons. Enumeration data were analyzed using chi-square tests and presented as frequencies and percentages. Prior to performing parametric analyses, assumptions about normality and homogeneity of variance were tested using the Shapiro–Wilk test and Levene’s test, respectively. The repeated measures analysis of variance (ANOVA) was used over alternative models, such as linear mixed models, because it specifically accounts for the within-subject correlations over multiple time points, which is more appropriate for the longitudinal data structure of this study. Potential confounding factors, including comorbidities, were controlled through the inclusion criteria, which required patients to be classified as ASA Grade I or II, ensuring that only patients with a relatively low risk of comorbidities were included. Additionally, statistical adjustments for potential confounders were made during analysis, where appropriate. A *post-hoc* power analysis was conducted to confirm the adequacy of the sample size and ensure that the study had sufficient statistical power to detect meaningful differences between the groups. A *p*-value of <0.05 was considered statistically significant.

## 3 Results

### 3.1 Comparison of general data

A total of 106 participants were included in the study, with 53 patients assigned to the intervention group and 53 to the control group. A comparison of baseline characteristics between the two groups revealed no statistically significant differences (*p* > 0.05), indicating that the groups were comparable. The findings of this study indicated that integrating the constructivist-based anesthesia education model into routine clinical protocols could significantly enhance preoperative patient education. This could involve incorporating multimedia tools, such as micro-videos and interactive simulation-based training, within standard anesthesia workflows, ensuring that patient anxiety is reduced and knowledge retention is maximized. These baseline characteristics confirmed the comparability of both groups, reinforcing the reliability of subsequent findings in relation to the intervention’s effects. Detailed results are presented in Table 1.

**Table 1:** Comparison of general data between the two groups.

Item	Control group (*n* = 53)	Intervention group (*n* = 53)	Statistic value	*p*-value	95% CI
Sex [*n* (%)]					
Male	21 (39.6%)	24 (45.3%)	*χ*^2^ = 0.348	0.556	–
Female	32 (60.4%)	29 (54.7%)			
Age (years, x̄ ± s)	46.0 ± 9.53	45.1 ± 9.40	*t* = 0.492	0.623	(−2.013, 2.573)
BMI (x̄ ± s)	24.3 ± 2.89	24.1 ± 1.77	*t* = 0.276	0.783	(−0.839, 1.069)
Educational level [*n* (%)]					
Primary school or below	6 (11.3%)	6 (11.3%)	*χ*^2^ = 4.531	0.210	–
Junior high	21 (39.6%)	12 (22.6%)			
Senior high	15 (28.3%)	24 (45.3%)			
Undergraduate or above	11 (20.8%)	11 (20.8%)			
Occupation [*n* (%)]					
Worker	4 (7.5%)	5 (9.4%)	*χ*^2^ = 4.882	0.300	–
Farmer	13 (24.5%)	15 (28.3%)			
Teacher	10 (18.9%)	16 (30.2%)			
Staff	12 (22.6%)	11 (20.8%)			
Other	14 (26.4%)	6 (11.3%)			
Preoperative 1d blood pressure					
SBP (mm Hg, x̄ ± s)	125.49 ± 13.34	125.81 ± 10.66	*t* = 0.137	0.892	(−4.314, 4.140)
DBP (mm Hg, x̄ ± s)	80.49 ± 10.59	78.51 ± 9.30	*t* = 1.023	0.309	(−1.446, 3.465)
HR (x̄ ± s)	75.87 ± 10.05	77.26 ± 8.25	*t* = 0.781	0.436	(−3.799, 2.418)

(1) *t*-value; (2) *χ*^2^-value.

### 3.2 Comparison of SAI scores between the two groups before and after intervention

Before the intervention, there was no significant difference in the SAI scores between the two groups (*p* = 0.669, 95% CI: −4.342, 5.012), indicating no baseline difference. After the intervention, the intervention group showed significantly lower SAI scores compared to the control group (*p* = 0.024, 95% CI: −13.349, −0.489), indicating that the constructivism-based anesthesia education program effectively reduced preoperative anxiety. These results confirmed the effectiveness of the constructivism-based education model in reducing preoperative anxiety, demonstrating significant improvements in patient outcomes after the intervention. Detailed results are shown in Table 2.

**Table 2:** Comparison of SAI scores between the two groups before and after intervention (scores, x̄ ± s).

Group	Number of cases	SAI pre-intervention	SAI post-intervention	*t*	*p*-value	95% CI
Control group	53	48.11 ± 6.55	41.64 ± 7.55	*t* = 0.429	0.669	(−3.457, 3.954)
Intervention group	53	48.58 ± 4.59	39.06 ± 3.08	*t* = 2.307	0.024	(−13.349, −0.489)

### 3.3 Comparison of anesthesia health knowledge questionnaire scores between the two groups

The intervention group had higher anesthesia health knowledge scores than the control group, and the difference in scores was statistically significant (*p* = 0.002, 95% CI: 1.327, 10.373), as shown in Table 3. Higher anesthesia health knowledge scores in the intervention group indicated that the education model enhanced patient understanding of anesthesia, further supporting its efficacy in improving perioperative care.

**Table 3:** Comparison of anesthesia health knowledge questionnaire scores between the two groups (scores, x̄ ± s).

Group	Number of cases	Anesthesia health knowledge questionnaire score	*t*	*p*-value	95% CI
Control group	53	81.37 ± 11.66	*t* = 3.210	0.002	(1.327, 10.373)
Intervention group	53	88.21 ± 10.23			

### 3.4 Comparison of biological indicator scores between the two groups

There were no significant differences in biological indicators (SBP, DBP, and HR) between the two groups on the day before surgery (*p* > 0.05). However, upon entering the operating room and before extubation, the intervention group exhibited significantly more stable SBP, DBP, and HR compared to the control group (*p* < 0.05). After extubation, SBP and DBP differences were not statistically significant (*p* > 0.05), which may be partly due to the effects of sedation or analgesia administered to all patients at this stage. However, HR remained significantly different between the groups (*p* < 0.05). The stable physiological indicators in the intervention group before extubation, alongside the maintained difference in HR post-extubation, indicated that the education model not only alleviated anxiety but also contributed to better physiological regulation. The persistence of a significantly lower HR in the intervention group post-extubation, while SBP and DBP normalized, could be attributed to the different physiological mechanisms underlying HR regulation. While blood pressure often stabilizes quickly after sedation and extubation, HR is more sensitive to emotional and psychological factors, such as preoperative anxiety, which could be better managed by the intervention. This indicates that the education model may have a lasting impact on autonomic regulation even after the immediate effects of anesthesia. Detailed results are shown in Table 4.

**Table 4:** Comparison of biological indicator scores between the two groups (scores, x̄ ± s).

Indicator	Control group (*n* = 53)	Intervention group (*n* = 53)	*t*	*p*-value	95% CI
SBP (mm Hg)					
1 day before surgery	125.49 ± 13.34	125.81 ± 10.66	*t* = 0.137	0.892	(−4.314, 4.140)
Upon entering the operating room	134.79 ± 12.55	129.02 ± 9.77	*t* = 2.643	0.009	(0.724, 8.239)
Before extubation	128.81 ± 9.20	122.40 ± 11.06	*t* = 3.247	0.002	(2.205, 10.819)
DBP (mm Hg)					
1 day before surgery	80.49 ± 10.59	78.51 ± 9.30	*t* = 1.023	0.309	(−1.446, 3.465)
Upon entering the operating room	134.79 ± 12.55	129.02 ± 9.77	*t* = 2.855	0.005	(1.416, 8.574)
Before extubation	128.81 ± 9.20	122.40 ± 11.06	*t* = 2.805	0.006	(2.195, 9.755)
HR (bpm)					
1 day before surgery	75.87 ± 10.05	77.26 ± 8.25	*t* = 0.781	0.436	(−3.799, 2.418)
Upon entering the operating room	86.36 ± 8.25	81.89 ± 7.68	*t* = 2.888	0.005	(1.091, 7.889)
Before extubation	84.79 ± 11.46	78.55 ± 10.31	*t* = 2.950	0.004	(2.181, 8.915)

*F*-values, *p*-values for between-group, within-group, and interaction effects are also provided as statistical summaries.

## 4 Discussion

### 4.1 The constructivism learning theory-based anesthesia health education model demonstrates scientific guidance for laparoscopic cholecystectomy patients

The primary objective of this study was to assess the impact of a constructivism learning theory-based anesthesia health education program on preoperative anxiety and anesthesia-related knowledge in laparoscopic cholecystectomy patients. The findings offer compelling evidence that this approach enhances both emotional and cognitive outcomes in this patient population. The constructivism learning theory-based anesthesia health education model provides an innovative and effective preoperative guidance approach for laparoscopic cholecystectomy patients. Rooted in the principles of active knowledge construction and contextualized collaboration, this model enables patients to internalize information through interactive learning experiences and communication (11). Previous studies have shown that surgical patients not only require factual preoperative information but also seek insight into the experiential aspects of their procedures. Building on this basis, the present study integrates perioperative experiences and the emotional needs of laparoscopic cholecystectomy patients into the anesthesia education framework.

Using a micro-video developed from the patient’s perspective, the intervention provided a peer-style educational experience, allowing patients to visualize peri-anesthesia scenarios chronologically (e.g., visual, auditory, and sensory aspects). This was further reinforced by anesthesia nurses through simulation-based training, helping patients master anesthesia cooperation strategies. The model’s focus on active patient participation ensures that education is individualized, addressing the unique needs of each patient while fostering knowledge construction through guided simulations and reflective teach-back sessions.

The statistically significant improvement in SAI scores and anesthesia health knowledge suggest that the constructivism-based model promoted active knowledge development, as evidenced by higher knowledge retention and reduced anxiety. These outcomes reflect the key principles of constructivism, particularly in how knowledge is built through active, patient-centered learning experiences, and how anxiety is alleviated through familiarization and engagement with the learning content.

Considering that 40–80% of medical information is forgotten almost immediately, with nearly half of the retained information being inaccurate (12), this model’s teach-back method plays a major role in improving knowledge retention and refining the educational process. For laparoscopic cholecystectomy patients, this approach not only enhances their understanding of surgical and anesthesia processes but also boosts confidence and compliance. The model creates a dynamic, scientifically guided educational framework by integrating psychological and emotional needs with educational strategies and outcome evaluation.

### 4.2 Constructivism theory-based anesthesia education improves peri-anesthesia experiences and alleviates preoperative anxiety in laparoscopic cholecystectomy patients

Hospitalized patients often face stressors, such as disease burden, unfamiliar environments, and surgical uncertainties, all of which contribute to heightened psychological distress. Studies show that 46.8% of surgical patients experience preoperative anxiety (3), with anxiety levels increasing as the surgery date approaches (2). Preoperative anxiety is known to elevate blood pressure and HR, jeopardizing surgical safety, increasing anesthetic and analgesic requirements, and impairing recovery (13). Severe anxiety can also suppress immune function, increasing infection risks and delaying wound healing.

This study demonstrated that the constructivism learning theory-based anesthesia education model significantly reduced patients’ SAI scores after the intervention, which suggests that the model effectively alleviated perioperative anxiety in laparoscopic cholecystectomy patients. In addition, the intervention group scored higher on anesthesia health knowledge questionnaires than the control group, indicating improved understanding and mastery of anesthesia-related information. Each component of the intervention was specifically designed to align with core constructivist elements. For example, the micro-video used visual and sensory cues to provide contextualized, real-world experiences, fostering active engagement and experiential learning. Interactive exercises, such as practicing anesthesia cooperation points, promoted hands-on learning, while the teach-back method ensured active reflection, reinforcing knowledge construction and self-assessment.

Previous studies indicate that patients often feel a loss of control upon entering the operating room, exacerbating their psychological vulnerability (14). Pre-familiarizing patients with the operating room environment and anesthesia workflows reduced physiological stress markers (SBP, DBP, HR) during operating room admission and pre-extubation. However, the lack of significant differences in post-extubation blood pressure between groups may be attributed to residual sedation effects and delayed pain perception during early recovery. Recent studies (14) have also highlighted the effectiveness of constructivist learning approaches in medical education, particularly in reducing patient anxiety and improving knowledge retention in surgical contexts. These studies further support the findings of the current research, suggesting that constructivism-based education can play a key role in enhancing patient experiences and outcomes. The lack of significant differences in post-extubation blood pressure between groups may be attributed to residual sedation effects and delayed pain perception during early recovery. Notably, HR remained significantly lower in the intervention group, which may reflect the psychophysiological effects of reduced preoperative anxiety. Lower HR post-extubation could be linked to the enhanced relaxation and emotional regulation promoted by the constructivist-based education model. This model’s concentration on alleviating anxiety and stress through preoperative familiarization and experiential learning may contribute to a more controlled autonomic nervous system response during recovery, promoting a more stable cardiovascular profile.

This study addressed a critical gap in the current literature by exploring the application of constructivism-based anesthesia education in the perioperative setting. While most existing research has focused on general patient education, few studies have addressed the emotional and cognitive aspects of anesthesia education for surgical patients. This approach provides a novel and comprehensive solution to improve both psychological and physiological outcomes in anesthesia-related education. These findings highlight the effectiveness of the constructivism-based education model in reducing preoperative anxiety and improving physiological and emotional well-being throughout the perioperative period. By addressing both psychological and physiological stressors, the model provides a comprehensive approach to enhancing peri-anesthesia experiences for laparoscopic cholecystectomy patients. To promote broader clinical adoption of the constructivism-based anesthesia education model, future research should concentrate on nurse training and the development of an educational toolkit to ensure consistent implementation across healthcare settings. Providing healthcare professionals with standardized resources and training programs may allow for seamless integration of this model into routine clinical practice. Additionally, feasibility studies should assess how this approach can be adapted to different hospital settings, including high- and low-resource environments, to evaluate its applicability and scalability. While this study primarily concentrated on short-term physiological outcomes, future research should consider assessing longer-term outcomes, such as chronic pain and psychological distress, to evaluate the sustained benefits of anesthesia health education. Specifically, follow-up assessments beyond the immediate post-surgical period (e.g., 3 months, 6 months, or longer) could provide valuable insights into the lasting effects of the intervention on patient recovery and wellbeing. Chronic pain and psychological distress, as important components of postoperative recovery, may help in understanding the broader impact of education on patients’ long-term health outcomes.

The results of this study contribute to the growing body of evidence supporting the effectiveness of constructivism-based anesthesia education programs in improving patient outcomes. Previous studies have explored the use of educational interventions to reduce preoperative anxiety and improve knowledge retention; however, the use of constructivist principles in anesthesia education has been less widely investigated. In comparison to earlier studies that used more traditional education methods, findings of the present study demonstrated that integrating multimedia tools, such as micro-videos and interactive simulations, into routine clinical protocols could significantly enhance both preoperative education and patient anxiety management. Specifically, this study showed a significant reduction in SAI scores and improved anesthesia health knowledge in the intervention group, aligning with findings from previous research, while providing new insights into the potential long-term benefits of constructivist learning (15). Moreover, this study uniquely highlighted the physiological implications of preoperative education, highlighting that patients in the intervention group not only exhibited reduced anxiety but also demonstrated more stable physiological indicators, including blood pressure and HR, before extubation. This differs from previous research that primarily concentrated on psychological outcomes and suggests that such educational interventions may have a broader impact, influencing autonomic regulation and overall patient physiology. These findings advance the current understanding by providing evidence that constructivist-based anesthesia education can play a significant role in improving perioperative care, reducing anxiety, and enhancing knowledge retention, while also promoting more stable physiological responses during the surgical process. This approach can be particularly beneficial in settings where patients experience high levels of preoperative anxiety or where there is a need for improved patient cooperation during anesthesia induction and recovery. Furthermore, while previous studies have primarily used general education strategies, the present study emphasized the importance of incorporating active, participatory learning experiences, which have been shown to have a superior effect on both anxiety reduction and knowledge acquisition. These findings may have important clinical implications, particularly in improving patient satisfaction and outcomes in anesthesia practice.

Despite the promising results, this study has several limitations. One potential limitation is selection bias, as patients who were selected to participate in this study might be more motivated or less anxious than the general surgical population, which could affect the generalizability of the findings. Additionally, the study was conducted at a single center, which might limit the ability to extrapolate results to diverse hospital settings. Another limitation is the lack of long-term follow-up to assess whether the benefits found in anxiety reduction and knowledge retention persist over time, hindering us from assessing the sustained effects of the anesthesia health education program on patient outcomes, such as long-term anxiety reduction, postoperative complications, or quality of life. Future research should aim to include longer follow-up periods to evaluate the sustainability of the intervention’s effects and explore whether the model has enduring impacts on postoperative outcomes, such as recovery speed and patient satisfaction.

In summary, the constructivism learning theory-based anesthesia education model offers a comprehensive, effective approach to reducing preoperative anxiety and improving anesthesia-related knowledge in laparoscopic cholecystectomy patients. By addressing both the cognitive and emotional needs of patients, this model enhances perioperative experiences and prepares patients for surgical procedures more effectively. Future studies should aim to explore its long-term benefits and applicability in diverse clinical settings, as well as the potential for expanding this model to other surgical specialties. Further research is also needed to evaluate the feasibility of integrating this educational approach into routine practice across various healthcare environments, ensuring its widespread adoption and impact on patient outcomes.

## 5 Conclusion

The constructivism learning theory-based anesthesia education model has a positive impact on clinical outcomes for laparoscopic cholecystectomy patients. This model enhances the overall perioperative experience by increasing anesthesia knowledge, alleviating preoperative anxiety, and promoting effective collaboration between patients and nurses. Through participatory and interactive educational methods, patients gain a deeper understanding of surgical and anesthesia processes, effectively alleviating anxiety during preoperative preparation, operating room admission, and anesthesia recovery. This approach improves perioperative experiences while emphasizing the significance of constructivism-based education in modern perioperative care. Key components of the constructivist learning theory, such as active learning and contextualization, were central to the outcomes observed in this study. By engaging patients in interactive, participatory education, the model allowed for a deeper internalization of anesthesia knowledge. Contextualizing information within real perioperative scenarios and fostering collaborative learning helped alleviate anxiety and enhance patient cooperation, reinforcing the constructivist principles of knowledge construction and meaningful engagement with the content.

The model demonstrates potential for broader application in various surgical contexts beyond laparoscopic cholecystectomy. Given its positive impact on preoperative anxiety reduction, enhanced knowledge acquisition, and improved patient cooperation, this model could be adapted for other types of surgery that require general anesthesia. For example, it may be effective in surgeries, such as orthopedic, cardiac, or gastrointestinal procedures, where patients’ understanding of anesthesia procedures and anxiety management are also critical to successful outcomes. These patient populations, often facing higher levels of anxiety or less understanding of the surgical process, could particularly benefit from the model’s personalized, constructivist approach. Further research in these areas could help establish the model’s versatility and confirm its effectiveness across diverse patient populations and surgical settings. By refining and adapting the educational content to fit the specific needs of different surgeries, the model could contribute to improving the overall quality of perioperative care in a wide range of clinical environments. To implement this model in clinical practice, hospitals and nursing educators can begin by integrating the constructivist learning approach into preoperative education protocols. This could involve training nursing staff in interactive teaching strategies and providing patients with tailored educational resources, such as micro-videos and simulation-based training, to enhance their understanding of the anesthesia process and reduce anxiety.

Ultimately, integrating theory-driven education, such as the constructivist-based anesthesia model, into national perioperative guidelines could revolutionize patient care by ensuring that all patients receive the benefits of personalized, interactive education. This would not only enhance patient outcomes but also contribute to the advancement of evidence-based practices in perioperative nursing.

## Data Availability

The original contributions presented in the study are included in the article/supplementary material, further inquiries can be directed to the corresponding author.
